# Physical and Chemical Stability of Mycophenolate Mofetil (MMF) Suspension Prepared at the Hospital

**Published:** 2012

**Authors:** Fanak Fahimi, Shadi Baniasadi, Seyed Alireza Mortazavi, Hanie Dehghan, Afshin Zarghi

**Affiliations:** a *Clinical Pharmacy Department, School of Pharmacy, Shahid Beheshti University of Medical Sciences*; b *Chronic Respiratory Disease Research Center (CRDRC), NRITLD, Masih Daneshvari Hospital, Shahid Beheshti University of Medical Sciences.*; c *Pharmaceutical Care Department, Virology Research Center, NRITLD, Masih Daneshvari Hospital, Shahid Beheshti University of Medical Sciences, Tehran, Iran*; d *Pharmaceutics Department, School of Pharmacy, Shahid Beheshti University of Medical Sciences, Tehran, Iran.*; e *Medicinal Chemistry Department, School of Pharmacy, Shahid Beheshti University of Medical Sciences, Tehran, Iran.*

**Keywords:** Extemporaneous pharmacy, HPLC, Mycophenolate mofetil, Stability, Suspension

## Abstract

To evaluate the physical and chemical stability of a suspension of mycophenolate mofetil (MMF) prepared in the hospital from commercially available MMF capsules and tablets.

Extemporaneous pharmacy was used as a feasible method in this experimental study to prepare suspension form of MMF. Suspension formulations were prepared from both tablets and capsules forms of MMF. Thereafter the stability parameters such as pH, microbial control, thermal and physical stability and particle sizes were evaluated. The amount of MMF, in the suspension was measured at various time points by HPLC.

The HPLC method showed that concentration of suspensions prepared from tablets and capsules were 49 mg/mL and 50 mg/mL at time 0, respectively. The effective amount of suspensions prepared from capsules was 101% at time 0, 100% after 7 days, 98% after 14 days, and less than 70% after 28 days.

According to the obtained results in this study, capsule-based suspension was stable for as long as 14 days at 5**°**C. This formulation appears to be clinically acceptable and provides a convenient dosage form for pediatric patients and for adults during the early postoperative period.

## Introduction

MMF is the morpholinoethyl ester of mycophenolic acid, which was originally isolated in 1896 from a penicillium culture (1, 2). After beneficial effects were observed in animals, Sollinger *et al*. (3) conducted the first human trials in kidney transplant recipients. MMF is used in patients who have recived kidney transplants and other solid organ transplants (4, 5). A prodrug, MMF is rapidly hydrolyzed to mycophenolic acid after oral administration. Mycophenolic acid inhibits the novo purine synthesis, resulting in antiproliferative effects on T and B lymphocytes (6). MMF is rapidly absorbed after oral administration, there is quick presystemic conversion to mycophenolic acid (the active metabolite) (7). 

The lack of commercially available oral liquid dosage forms is an ongoing problem in many practice settings. A pharmacist is often challenged to provide an extemporaneous oral liquid for (i) paediatric patients; (ii) patients who are unable to swallow solid dosage forms such as tablets or capsules; (iii) patients who must receive medications via nasogastric or gastrostomy tubes; and (iv) patients who require non-standard doses that are more easily and accurately measured by using a liquid formulation (8).

For oral administration, MMF is available as capsules 250 mg and tablets 500 mg. The suspension form, 200 mg/mL (Roche Products Ltd, UK), is not available and manufactured in Iran but is available in other countries. Considering low demands for this product, the high price of it ($299), and toxicity of the crushed tablets (9) necessitate pharmacist involvement to retain compounding skills. 

## Experimental

*Preparation of the suspension*

MMF suspension (50 mg/mL) was prepared in a vertical flow hood. The contents of six capsule (250 mg each)/or three tablets (500 mg each) were emptied into a mortar, wetted and triturated with Ora-Plus (7.5 mL) to a smooth paste. Fifteen mL of simple syrup was added and further triturated. The contents were then transferred into an amber bottle and made to a final volume of 30 mL with simple syrup (6). Then cherry essence was added. The cherry flavor helps masking the drug’s bitter taste (10) (Table 1). 

**Table 1:** Components for suspension preparation from MMF tablets/capsules

**Component**	
**MMF ** **Ora-plus®** **Simple syrup ** **Methyl paraben ** **Propyl paraben ** **Essence ** **Aspartam**	Three Tablets or Six capsules 7.5 mL 15 mL 0.054 mg 0.006 mg 1 drop q.s

Ora-plus was prepared in the laboratory (Table 2)

**Table 2:** Components for Ora-plus®

**Ingredient % **	
Water	97
Sodium phosphate monobasic	1≤
Carboxy methylcellulose Sodium	1≤
Microcrystalline cellulose	1≤
Xanthan gum	1≤
Carrageenan	1≤

BP (British Pharmacopoeia) grade syrup was used, which contained 667 g sucrose, purified water to 1,000 mL, and one or more suitable antimicrobial preservatives were added (5,9). For pH adjustment monobasic dihydrogen sodium was used and pH adjusted in range of 5-6. Long time and short time stability were evaluated (11). In the first study, the suspensions in amber containers were periodically maintained at 5 **°**C for 24 h and 40 **°**C (in an oven) for 24 h for 1 week. The formulated MMF suspensions were then stored over a period of 50 days at 5, 25 and 40**°**C. The samples were exposed to room light only. Particle size for suspensions that were prepared from tablets were evaluated by Mastersizer instrument. Trypticafe Soy Agar and Mannitol Salt Agar were used for microbial evaluation. 

*Analysis*

**Figure 1:**
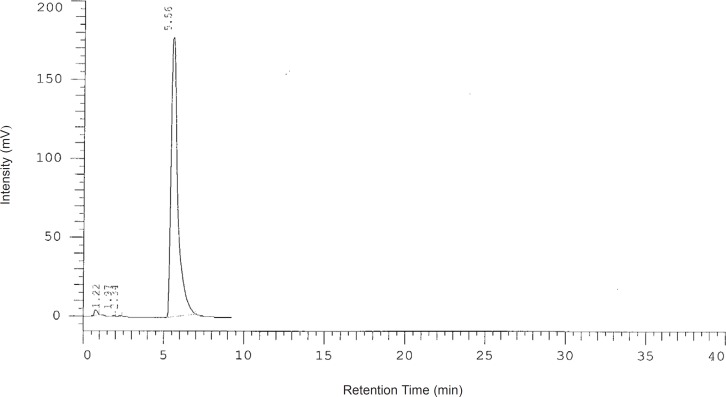
A typical chromatogram of MMF in a aqueous solution

The concentration of suspension was measured by a modified HPLC method. The analysis was achieved on C₁₈ Column (125X 4.6 mm, 5 μm) using acetonitrile-water (42:58 v/v), pH 3.5, as the mobile phase at a flow rate of 1.3 mL/min. The wavelength was set at 219 nm. A typical chromatogram of MMF in a aqueous solution is shown in Figure 1. The concentrations were evaluated at 0, 7, 14 and 28 days after preparation.

## Results

The concentration of suspensions prepared from tablets and capsules were 49 mg/mL and 50 mg/mL right after preparation (Time 0), respectively. The effective amount of suspensions prepared from capsules was 101% at time 0. After one week of observation, the color and the appearance of the suspension did not change at 5, 25, 37 and 45**°**C. Over 50 days of observation, the color and the appearance of the suspension did not change at 5, 25**°**C but the suspension became darker in 40**°**C. Monitoring particle size for suspension made from tablet showed a coarser suspension. The largest size was 79.433 μm. All microbial cultures were negative. 

The effective amount of suspensions prepared from capsules was 101% at time 0, 100% after 7 days, 98% after 14 days, and less than 70% after 28 days.

## Discussion

We prepared a suspension from the commercially available capsule and tablet, and evaluated its physical and chemical stability. Ora-plus (suspending agent) is not available in Iran. Ora-plus was prepared in the laboratory. We used the simple formulation for preparing oral suspension of MMF and its suspending agent. This formulation enables preparation of MMF suspension where and when needed in the pharmacy. Both tablets and capsules of MMF are always available in Iran. We made oral suspension of MMF from both tablets and capsules. Extemporaneously formulating, the suspension stability was acceptable in both suspensions prepared from capsules or tablets on the first day of preparation.

If MMF suspension would be used beyond 24 h after preparing, it is recommended to use it within 7 days. After 7 days the suspensions made from tablets were not in optimal concentration according to HPLC method. Based on reference when there is no data on stability for an extemporaneously formulated product as oral suspension, one could assume it has 14 days beyond use date in refrigerator (12). Interestingly, according to our finding this is not suitable for MMF prepared from tablets. This finding is of great importance since an organ transplanted patient may need MMF as suspension and the risk associated with a degraded dosage form could harm the patient in a vast extent. Many NPO (nothing by mouth) patients may need oral suspension of MMF and it can be prepared daily for them. But the pH, physical conditions, thermal stability, and appearance all were satisfactory. The suspensions made from capsules showed a relatively near optimal concentration after 14 days.

Besides, preparing the product extemporaneously is more cost benefit. Many orders would soon change from NPO to PO which will enable patient to receive solid forms of medications. Each bottle of commercially available suspension by Roche is $299 per 175 mL and by changing order the rest of the unused drug will be discarded. While, we can make small volumes *e.g. *30 mL at Pharmacy. This will help to cut unnecessary expenses.

Comparing tablet and capsule based suspensions showed no difference between suspensions regarding appearance, physical and thermal stability. But after 14 days the concentration and total stability were more appropriate in those prepared from capsules.

In a study by Venkataraman *et al. *(10) performed in 1998 the MMF suspension was prepared by EP method. They prepared suspension with concentration of 50 mg/mL and used HPLC method. The thermal stability was evaluated in 5 and 25**°**C. The suspension had a half-life of 98 days at pH 2, 118 days at pH 5.1 and 19 days at pH 7.4. Therefore, we kept the pH in a range of 5-6.

Another study by Anaizi *et al. *(11) evaluated the stability of oral preparation of MMF. The concentration of the preparation was 100 mg/mL with a volume of 200 mL and stored in polyethylene tetraphthalate glycol bottles. They also used HPLC method. The final pH was 6.1 that kept up to final stages. There was no color change and no positive microbial culture. After 121 days a ten percent reduction in final concentration of suspension was reported in their study.

Extemporaneous pharmacy is considered as an important service performed by pharmacists in the clinical sites. Extemporaneous compounding is a big challenge in developing countries. Since pharmacies are generally not very well equipped to carry out extemporaneous compounding of such formulations and standards in developing countries will be limited by lack of resources (including the availability of active ingredients and even water or equipment), trained personnel and facilities (13).

Pharmacists are responsible for ensuring that drug use is safe and effective. Wherever practicable, licensed medicines are used and represent the ‘gold standard’ for quality, safety and efficacy. There are, however, circumstances in which a medicine (such as MMF suspension) that fully meets the clinical needs of a particular patient or patients is not available (14). 

Sometimes patients and nurses are not aware that crushing tablets or opening capsules may cause toxicity or instability of the products. It is pharmacist responsibility to educate and consult patients and nurses regarding proper use of medicines and extemporaneously prepare a limited quantity of a custom-made medicine for a specific patient (15). 

Our study can be a practical model for other developing countries where pharmacists should be more familiar with different aspects of their profession. 

The clinical pharmacy program was designed in our hospital to provide an opportunity for pharmacy students to attain education (14). Nonsterile compounding is considered as one of the new services defined in Masih Daneshvari clinical pharmacy program.

In this study, we prepared Ora-plus in the laboratory environment while in all mentioned studies commercial Ora-plus® was used. This may explain for the less stable MMF oral suspension and the probable variations of pH. 

So, in conclusion compounding MMF suspension from capsules in the pharmacy and reusing prepared bottles when the order is changed without concern for stability of the solution for at least 14 days at 5 ºC is possible.
